# Innominate Artery Compression Syndrome

**DOI:** 10.1055/s-0042-1742697

**Published:** 2022-11-01

**Authors:** Achala Donuru, Vinay Kandula, Peace Madueme

**Affiliations:** 1 Department of Radiology, Thomas Jefferson University Hospitals, Philadelphia, Pennsylvania; 2 Department of Medical Imaging, Nemours Children's Hospital, Wilmington, Delaware; 3 Department of Cardiology, Nemours Children's Hospital, Orlando, Florida

**Keywords:** innominate artery, trachea, compression

## Abstract

Vascular mediated airway compromise is a fairly common clinical scenario. The diagnosis of innominate artery compression may be challenging due to lack of standardized imaging criteria for diagnosis or for surgical intervention.

A 2-month-old boy presented with noisy breathing and dry cough from 3 weeks of age. Symptoms were worsening with time. A preliminary clinical diagnosis of laryngomalacia was made. Chest radiograph showed marked narrowing of the intrathoracic trachea in the anteroposterior (AP) dimension ( Fig. 1 ). He was evaluated by an ear, nose, and throat (ENT) surgeon. Rigid bronchoscopy revealed tracheal compression and tracheomalacia. Computed tomography (CT) angiography was performed, revealing significant compression of the upper intrathoracic trachea in the AP dimension where the innominate artery courses anterior to the trachea and to the right at the level of the thoracic inlet (axial CT, Fig. 2 , and three-dimensional volume rendered CT images, Figs. 3 and 4 ). Overall appearance was consistent with innominate artery compression syndrome. The patient was evaluated by the cardiothoracic surgery service and right anterior thoracotomy and aortopexy were performed. Postoperative bronchoscopy showed a much improved appearance of the trachea. The patient is doing well after surgery.

**Fig. 1:**
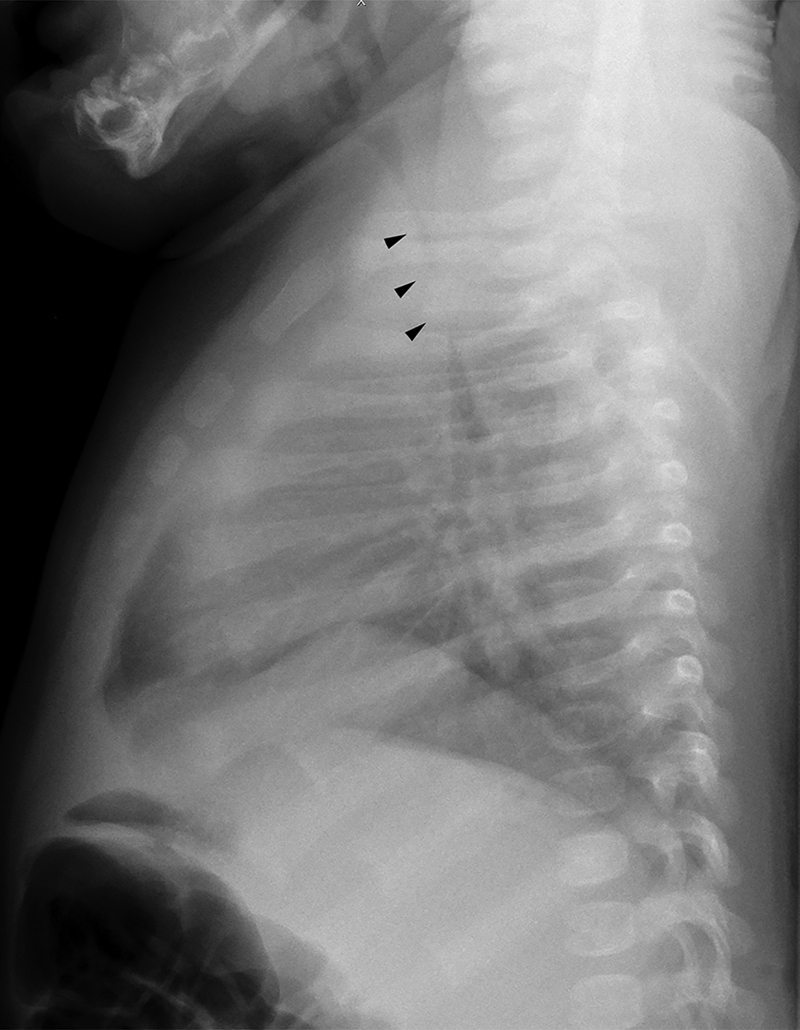
Lateral chest radiograph show tracheal narrowing (arrow heads).

**Fig. 2:**
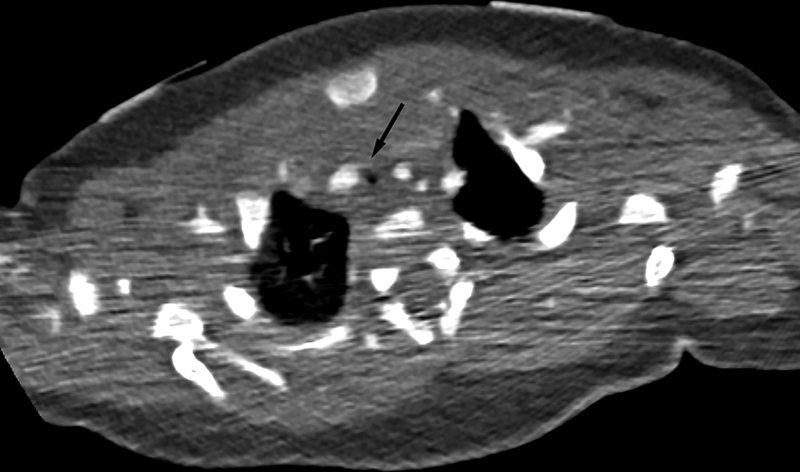
Axial chest computed tomography with intravenous contrast shows marked narrowing of the trachea (arrow) with the innominate artery to the right.

**Fig. 3:**
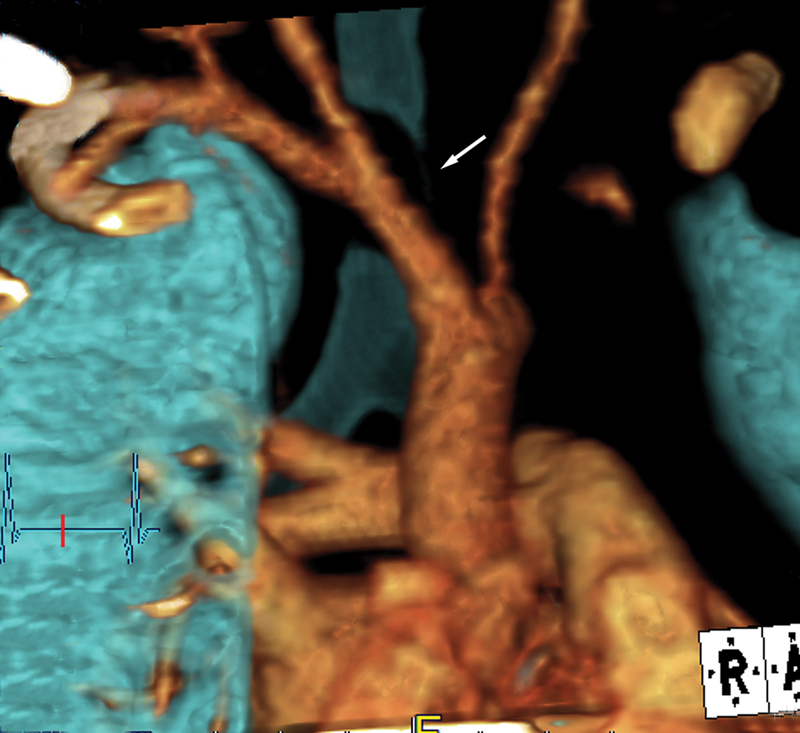
Three-dimensional volume rendered computed tomography image of the trachea demonstrates the innominate artery compressing the right lateral wall of the trachea (arrow).

**Fig. 4:**
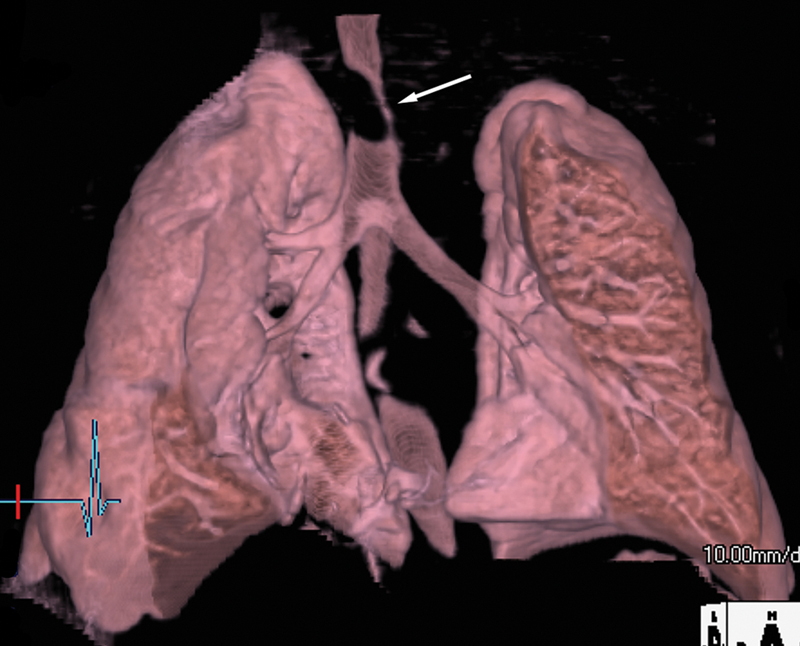
Three-dimensional volume rendered computed tomography image of the trachea demonstrates marked tracheal narrowing (arrow).

Common causes of stridor at birth include vocal cord paralysis, choanal atresia, laryngeal web, or vascular ring. Common causes of stridor during the first few weeks of life include laryngomalacia, tracheomalacia, and subglottic stenosis.

Tracheal compression by the innominate artery is the most common of the compression syndromes caused by incomplete vascular rings and a common causes of mechanical upper airway obstruction in children. 1 Brachiocephalic (innominate) artery compression syndrome occurs in the pediatric population, where the brachiocephalic artery usually takes its origin to the left of the trachea and subsequently compresses the trachea as it traverses anteriorly at the level of the thoracic inlet. This compression decreases with age and these patients commonly outgrow the narrowing.

Symptomatic patients typically present with expiratory stridor, cough, recurrent bronchopulmonary infections, and occasional apnea. 2 A lateral chest X-ray may show tracheal deviation or indentation or lung hyperinflation. CT and magnetic resonance imaging are useful in delineating the vascular anatomy in multiple planes. The majority of patients with innominate artery compression of the trachea are successfully treated with medical management. Medical management includes humidified oxygen, steroids, and antibiotics. Surgery is indicated for patients with apnea, multiple episodes of tracheobronchitis, or bronchopneumonia, and after 48 hours of failing to respond to medical therapy. 3 Aortopexy and innominate artery reimplantation are the most commonly performed procedures for innominate artery compression syndrome.
